# The Effects of War‐Related Stress on Human Development: Differences in Body Proportions of Polish Women Born Before and During World War II

**DOI:** 10.1002/ajhb.24175

**Published:** 2024-10-30

**Authors:** Linda Koníková, Grażyna Liczbińska, Miroslav Králík

**Affiliations:** ^1^ Department of Anthropology, Faculty of Science Masaryk University Brno Czech Republic; ^2^ The Institute of Human Biology and Evolution, Faculty of Biology Adam Mickiewicz University Poznań Poland

**Keywords:** anthropometry, human development, nutrition, stress, World War II

## Abstract

**Objectives:**

This study aims to explore the lasting effects of stress experienced by pregnant women during World War II (WWII) on body and head measurements of their adult daughters.

**Methods:**

The research sample consists of 336 female university students born in Poland between 1925 and 1951. The data include body measurements and socioeconomic information (parental occupation and number of siblings) acquired from questionnaires collected between the 1950s and 1970s. Student's *t*‐test, Mann–Whitney test and Analysis of Variance were used to analyze differences in body measurements between groups of women born before and during the war, as well as the possible influences of socioeconomic variables.

**Results:**

The mean measurements of body height, symphysion height, and waist circumference were lower in women conceived and born during the war compared to those born in the pre‐war period. In contrast, the mean measurements of biacromial (shoulder) width, trunk length, and three head dimensions were higher in women conceived and born during the war. Additionally, the number of siblings appeared to be a significant factor that may have influenced the body measurements of women in both groups. For instance, a higher number of living siblings, particularly sisters, was associated with reduced body dimensions, such as body height and waist circumference, while a greater number of deceased siblings was linked to an increase in certain body dimensions.

**Conclusion:**

The results suggest that war‐related prenatal conditions may have influenced the postnatal growth and development of women conceived and born during the war. Notably, the direction of these changes varied, which indicates that the growth response to the war‐related conditions was a complex adaptation, reflecting both positive and negative changes in different body parts, rather than a uniform pattern of growth suppression.

## 1 Introduction

External conditions, such as wars, natural disasters, political upheavals, or economic crises, which have indisputable impact on pregnant mothers, can have significant consequences for the development of offspring and their physical characteristics in later life. This has been confirmed by a large body of literature (e.g., Clarkin 2008, 2019; Dancause et al. 2011; Gomula et al. 2022; King et al. 2012; Lederman et al. 2004; Liczbińska, Piontek, and Malina 2017; Liczbińska et al. 2018, 2023; Liczbińska and Králík 2020; Palmeiro‐Silva et al. 2018; Xiong et al. 2008). One of the main factors influencing the intrauterine environment is stress experienced by pregnant mothers, which can lead to changes in hormonal balance in the body of mother and developing fetus. Outcomes of maternal stress are associated with changes in two major stress response systems: the hypothalamic–pituitary–adrenal axis and the sympathetic nervous system, both initiating a hormonal cascade resulting in a release of stress hormones, such as cortisol, which can cross the placenta and affect fetal development (Lazinski, Shea, and Steiner 2008; Ranabir and Reetu 2011).

In previous research, various sources of stress operating during pregnancy have been associated with various developmental outcomes in children. For example, shorter gestation period, lower birth weight and smaller head dimensions were linked to natural disaster‐related stress (Dancause et al. 2011; Gomula et al. 2022; King et al. 2012), as well as to the terrorist attack on the World Trade Center (Lederman et al. 2004). Research concerning war‐related stress has shown that women conceived and born during WWII were shorter and matured later compared to those born before and after WWII (Liczbińska, Piontek, and Malina 2017; Liczbińska et al. 2018, 2023). Furthermore, the relationship between prenatal exposure to political changes after WWII and later occurrence of menarche has been identified (Sekajová et al. 2022). Outcomes such as shortened gestational age, higher proportion of stillbirths and neonatal deaths, and earlier deliveries have been emphasized in connection to environmental stresses (Liczbińska and Králík 2020; Palmeiro‐Silva et al. 2018; Xiong et al. 2008). Moreover, elevated BMI with potential risk of diabetes, heart disease and stroke in later life was linked to maternal living conditions during pregnancy (Farewell et al. 2021; Leppert et al. 2018).

In addition, natural disasters, wars, and economic crises are not only sources of psychological stress, but they are also associated with food shortages and inappropriate nutrition, other important factors leading to changes in the intrauterine environment. The concept of fetal programming states that during the critical developmental window, an external stimulus initiates an adaptive response of the fetus, including changes in metabolism and hormone production, which may permanently affect the future structure, function or development of the organism and lead to maladaptation in later life (Barker 1995; Gluckman et al. 2008; Wells 2003). Importance of bi‐directional interaction between nutrition and stress was highlighted in the study of developmental plasticity (Entringer, Buss, and Wadhwa 2015). According to previous studies, more than 20% of variation in body height is a result of environmental factors connected mainly to socioeconomic background during childhood, but such factors as malnutrition may start to influence body height already during prenatal development (Silventoinen 2003).

Despite the wide scope of previous research, it is difficult to specify relationships between maternal stress during pregnancy and offspring characteristics in later life. Not all studies have found consistent effects since the observations might be influenced by a combination of various factors, including nutrition, socioeconomic status, living conditions, or historical period. Our study could, therefore, enhance understanding of the lasting effects of adverse historical events on human development.

### 1.1 Research Aims and Hypotheses

This study aims to explore the lasting effects of stress experienced by pregnant women with their daughters in uteruses during WWII by analyzing possible variations in adult body and head measurements of daughters born before and during the war. In other words, we aim to follow up the influence of prenatal factors related to the harsh conditions of war on postnatal growth and development.

We hypothesize that less energy may be allocated to growth due to a stressful environment and lack of nutrition, therefore women born during WWII are expected to have lower body measurements compared to those born before the war. Moreover, a significant relation between facial dimensions and the stressful wartime period may be expected. The human face, shaped by evolutionary selective forces as well as complex individual development, is an important source of biological (e.g., sex, age, health and hormonal status) and sociological information (e.g., social perception, interpersonal attitudes, and emotions), and may also reflect earlier environmental exposures of an individual. Moreover, we hypothesize that differences in body and head measurements resulting from the harsh conditions of war may be additionally influenced by the socioeconomic status of the family.

## 2 Materials and Methods

The anonymous database of 537 individuals (all female) born from 1925 to 1951 was established based on archival data. Male individuals were excluded from the analyses due to insufficient number of observations (*n* < 10 individuals). Women were subsequently divided into three groups based on their birth date in relation to WWII: (1) the “pre‐war group” born before September 1, 1939 (*n* = 240), (2) the “war group” born from September 1939 throughout 1945 (*n* = 249), and (3) the “post‐war group” born after 1946 (*n* = 48). The age of women at the time of survey was calculated based on their date of birth and date of survey ($\bar{x}$ = 21.74 ± 2.25 years). The material contained 21 anthropometric measurements. Body measurements included body weight (in grams), body height, tragion height, acromion height, suprasternal height, omphalion height, symphysion height, illiocristale height, dactylion height, biacromial width, and waist circumference (all in millimeters). Moreover, upper limb length was calculated by deducting dactylion height from acromion height and trunk length was calculated by deducting symphysion height from acromion height (all in millimeters). Head measurements included distances between anthropometric points: glabella–opisthocranion, euryon–euryon, mastoideale–mastoideale, frontotemporale–frontotemporale, zygion–zygion, gonion–gonion, nasion–gnathion, nasion–prosthion, nasion–subnasale, and alare–alare (all in millimeters). Moreover, index cephalicus was established as distance between euryon–euryon divided by distance between glabella–opisthocranion multiplied by 100. In total, 24 anthropometric measurements representing not just overall size, but also the proportions of specific body parts, were available and used to analyze how different body parts may differ in response to war‐related conditions.

Parental occupation and the number of siblings were included as variables describing socioeconomic status of women. Occupation of fathers and mothers was divided into four categories: (1) unemployed (27 fathers and 309 mothers), (2) white collar workers (232 fathers and 129 mothers), (3) blue collar workers (161 fathers and 21 mothers), and (4) military servicemen (16 fathers, 0 mothers). Factor “parental work” was created in accordance with previous categories (the category of father was used if both parents work) and three additional categories were assigned: both parents are without work (*n* = 13), one parent works (*n* = 263), both parents work (*n* = 131). Total number of siblings (min = 0, mean = 3, max = 13 siblings), which describes family size, included information about the number of siblings, and about their living status (alive or deceased). The category 4+ was created when the number of siblings alive was higher than four (i.e., four to five brothers and four to seven sisters). The category 6+ was created when the total number of siblings alive was higher than six (i.e., six to ten) and the category 3+ was created when the total number of deceased siblings was higher than three (i.e., three to nine).

The sample was inspected for missing variables within anthropometric measurements (NAs = 378, min = 1 in the distance between euryon–euryon, max = 106 in biacromial width). To preserve individual variation and group‐specific trends, observations containing missing variables in anthropometric measurements were omitted. Therefore, the number of observations was decreased to 195 in the pre‐war group and 174 in the war‐group. Only five observations without missing variables remained in the post‐war group. Data imputation would significantly skew the data distribution, and this category was therefore excluded from further analyses. To avoid possible errors during data collection and to receive a clearer picture of the variability within the majority of the data, outliers were removed from the dataset. To identify outliers, *z*‐scores were computed for each data point in the dataset. Based on data visualizations a threshold of 3 standard deviations was set to define the criteria for identifying extreme values. Data points with *z*‐scores greater than 3 or less than −3 were removed from the dataset. The number of observations was thereby decreased to final 336 individuals: 179 in the pre‐war group and 157 in the war group.

Student's *t*‐tests and Mann–Whitney tests were performed to analyze the effects of prenatal factors on body and head dimensions in adulthood. The analysis of variance (ANOVA) was performed for anthropometric measurements that significantly differed between groups. Linear regression models included anthropometric measurement as the dependent variable and several independent variables such as group and factors connected to parental occupation and number of siblings. The validity of a linear regression models was assessed using residual analysis. Line plots with a standard error of the mean (mean SE) were generated using ggplot2 (Wickham 2016), illustrating significant differences in anthropometric measurements within the groups. Mean SE was calculated as the sample standard deviation divided by the root of the sample size.

### 2.1 Ethics Statement

The material was collected by researchers from the former Department of Anthropology (currently Institute of Human Biology and Evolution), Adam Mickiewicz University in Poznań, between 1955 and 1972 as a part of the project aimed to analyze the biological characteristics of Polish students attending Adam Mickiewicz University in Poznań. Research data included social questionnaires and body measurements commonly used in anthropometric studies to describe body size and shape. Currently, these materials are deposited in the archives of the Institute of Human Biology and Evolution, AMU. The team had not applied to the Ethical Committee for permits for the research since in the 1950s and 1960s research procedures did not require it. Ethical committees have been operating in Poland only since the mid‐1990s. Nevertheless, all the procedures performed in studies involving human participants were in accordance with the internal (university) ethical standards. All participants were orally informed about the aims of the project and procedures of the study, and they had the opportunity to withdraw from the study at any time without giving any reason. All volunteers provided consent prior to their inclusion in the study. All procedures followed in the research were conducted in accordance with the ethical standards of the institutional and national research committee, and then with the 1964 Declaration of Helsinki. Personal data for this study were processed in accordance with Recitals 27, 158, and 150 and Article 89 of Regulation 2016/679 (GDPR), by which the processing of personal data of deceased persons is not covered by data protection provisions, however appropriate technical and organizational measures were applied to respect the rights of the participants (e.g., anonymization).

## 3 Results

**FIGURE 1:**
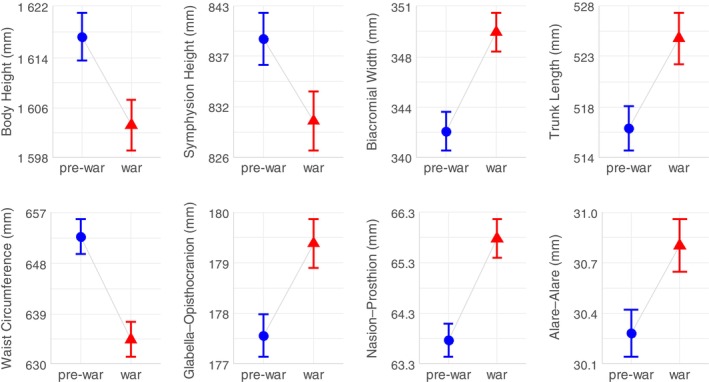
Significant differences in anthropometric measurements (in millimeters) among women from the pre‐war (blue color) and the war group (red color). Error bars indicate the standard error of the mean (SEM).

The results suggest that women in the war group differed significantly in various body and head measurements compared to women in the pre‐war group (Figure 1). Women in the war group had significantly lower mean measurements regarding body height (*t*‐test = 2.524, *p*‐value = 0.012), symphysion height (*t*‐test = 2.091, *p*‐value = 0.037), and waist circumference (*t*‐test = 4.174, *p*‐value = 0.000), and significantly higher mean measurements in biacromial width (*t*‐test = −3.616, *p*‐value = 0.000), trunk length (*t*‐test = −2.657, *p*‐value = 0.008), and head measurements including glabella–opisthocranion (*t*‐test = −2.848, *p*‐value = 0.005), nasion–prosthion (*t*‐test = −3.974, *p*‐value = 0.000), and alare–alare (*t*‐test = −2.492, *p*‐value = 0.013) compared with women in the pre‐war group.

**FIGURE 2:**
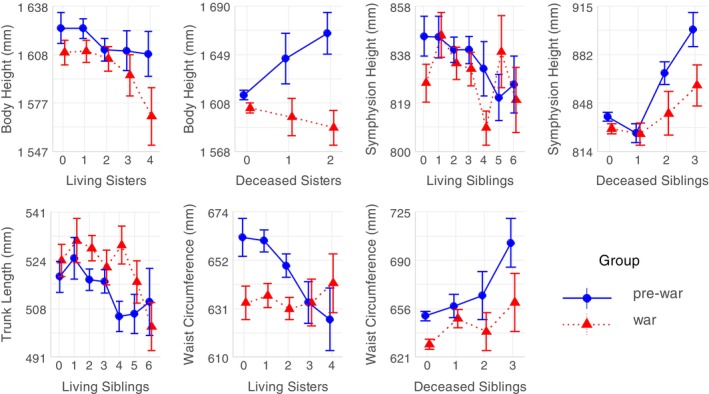
Significant differences in body measurements (in millimeters) among women from the pre‐war (blue color) and the war group (red color) by the number of living and deceased siblings. Error bars indicate the standard error of the mean (SEM).

Socioeconomic factors (parental occupation and number of siblings) did not significantly differ among women from the pre‐war and the war group. The number of siblings was a significant factor influencing anthropometric measurements of women in both groups (Figure 2 and Table S1). Firstly, as the number of living sisters increases, body height of women in the war group and waist circumference of women in the pre‐war group decrease. Second, as the number of living siblings increases, symphysion height and trunk length of women in the pre‐war group decrease. Additionally, as the number of deceased sisters increases, body height of women in the pre‐war group also increases. And finally, as the number of deceased siblings increases, symphysion height of women in the pre‐war group and waist circumference of women in both groups also increase. Parental occupation did not significantly affect the studied anthropometric measurements of women.

## 4 Discussion

We aimed to explore the connection between WWII and human body proportions and face dimensions, since wars are considered one of the strongest exogenous stressors in the human history. We hypothesized that stress experienced by pregnant women during wartime could have led to epigenetic changes influencing development and therefore physical characteristics of their daughters, which were studied in this research.

Based on our results, women from the war group had significantly lower mean measurements in body height, symphysion height and waist circumference. Body height strongly correlated with symphysion height, which can be considered a marker for the growth of lower limbs. Variation in body height can provide insight into nutritional and health status during development of an individual. Research has shown that early‐life malnutrition, particularly during critical periods such as pregnancy and early childhood, can impact growth and lead to shorter adult height (Moore 2023). Additionally, Schwab and Rakers (2023) noted that maternal psychosocial stress can further inhibit fetal growth, contributing to lower body measurements. Therefore, it is plausible that the combination of poor nutrition, war‐related stress, and associated maternal health challenges may have affected the growth in height of women from the war group. Furthermore, limited or disrupted access to food during wartime is likely linked to the significantly lower waist circumference observed in this group. Second, women from the war group had significantly higher mean measurements in biacromial width, trunk length, and certain head measurements, contrary to our hypothesis according to which we expected lower body and head measurements due to the stressful environment and lack of nutrition. These findings suggest that women from the war group may have undergone a complex adaptive response rather than a uniform pattern of growth suppression. Literature highlights that undernutrition and stress can lead to trade‐offs in growth patterns, where somebody dimensions may be preserved or enhanced at the expense of others (e.g., Godfrey and Barker 2001; Kuzawa 2005; Wells 2011). Therefore, it is possible that the observed increase in biacromial width, trunk length, and certain head measurements reflect an adaptive response to the war‐related stressors experienced during prenatal and early‐life development. Longer maximum head length (glabella–opisthocranion) may reflect an adaptive strategy that prioritizes the growth of crucial structures, such as the brain, even under adverse conditions like malnutrition and stress. This adaptation allows the brain to continue growing while other parts of the body may remain underdeveloped (Schwab and Rakers 2023; Wells 2011). Other head measurements, including distances between nasion–prosthion and alare–alare, may possibly indicate adaptive responses to harsh conditions and/or altered hormonal balances due to stressful environment. For instance, Butaric et al. (2021) explored ontogenetic variation in human nasal morphology, including aspects of sexual dimorphism, emphasizing how environmental factors can shape nasal development. Wider biacromial (shoulder) width reflecting upper body development may be increased as an adaptive response to harsh environment, prioritizing upper body strength (Kuzawa 2005) or/and it may be also linked to hormonal imbalance during fetal development caused by war‐related stress of pregnant mothers which could have led to blurred sexual dimorphism later in life. Similar results are indicated by the research on intergenerational transmission of stress where pregnancy of grandmothers during the World War I and the Great Depression blurred sexual dimorphism in the generation of their grandchildren (Liczbińska and Králík 2023). Trunk length showed opposite trend to body and symphysion height. Since women from the war group were overall shorter, their trunks were longer compared with the pre‐war group. It can be supposed that the timing of specific stress or hormonal exposure during certain critical periods may have impacted the development of particular body segments differently. In addition, the developmental plasticity of trunk may be lower since it contains vital organs which would also reflect trade‐offs in growth patterns—reallocated resources in response to stress (e.g., Godfrey and Barker 2001; Kuzawa 2005; Wells 2011).

The number of siblings appeared to be a significant factor that may have influenced body measurements of women in both groups. Figure 2 and Table S1 show that women from the pre‐war group with more siblings tend to have shorter symphysion height and trunk length. Similarly, women in the pre‐war group with more siblings, particularly sisters, tend to have lower waist circumference. Furthermore, symphysion height of women from the pre‐war group increased with the increase of the number of deceased siblings, predominantly deceased sisters. These relations may be interpreted as a result of resource dilution within a family (i.e., larger family size leads to fewer resources per member) and/or in connection with life history of mother and potential physiological burdens from earlier pregnancies (Blake 1981; King 2003). There was no significant relationship between the number of living (or deceased) siblings and previously listed measurements of women from the war group. We can hypothesize that war disrupted this effect because the resources were limited regardless of family size. Negative relationship was found between the number of living sisters and body height of women in the war group, that is, their body height increased along with the decrease of the number of living sisters, while in the pre‐war group, body height increased along with the increase of the number of deceased sisters, which may possibly reflect a selection of taller individuals (daughters). Finally, with higher number of deceased siblings, particularly deceased brothers, the waist circumference of women from both groups was higher since deceased siblings did not limit resources. In summary, resource dilution within a family may provide a partial explanation for the observed differences connected to the number of siblings (Blake 1981). However, complex interactions between various factors such as family size, sibling composition, maternal characteristics, and external factors like war should be considered. Parental occupation did not significantly influence body measurements observed in this study. In the harsh conditions of WWII socioeconomic status no longer played a role, which was demonstrated by earlier study (Liczbińska and Králík 2022).

## 5 Research Limitations

This work is not free of research limitations. First of all, our sample included only women. The initial research focused on students who attended the university in Poznań between 1955 and 1972, particularly those choosing teaching specialization. Female students dominated in the group, while fewer than 10 male students were present. Including such a small number of men would not have provided meaningful results and could have enhanced the complexity of the study since men have different hormone levels, growth dynamics, body composition, and life history. Therefore, our sample is not a random selection representative for the population. On the other hand, the homogeneity of this specific group may partially mitigate another limitation, which is that the studied relationship might have been influenced by a combination of various external factors such as environmental circumstances related to the war, living conditions (e.g., social status, availability of resources), or individual timing and intensity of the exposure to stressors. Second, we did not have data on parental biological characteristics, particularly the characteristics of mothers during pregnancy, such as body measurements, health and nutrition status or stress hormone levels, which might have potentially influenced the fetal development of their daughters. Third, we did not have information on the birth order of the studied daughters and their siblings. While the number of siblings was identified as a significant factor influencing body measurements, the absence of data on birth order may have confounded the results.

## 6 Conclusion

Various body dimensions differed significantly between women from the pre‐war and the war group, which suggests that prenatal factors related to wartime conditions could have influenced postnatal physical growth and development in women conceived and born during the war. Notably, the direction of these changes varied which indicates that the growth response to the war‐related conditions was a complex adaptation, reflecting both positive and negative changes in different body parts, rather than a uniform pattern of growth suppression.

## Author Contributions

**L.K.:** data curation, formal analysis, funding acquisition (lead), visualization (lead), writing – original draft preparation, writing – review and editing (equal). **G.L.:** conceptualization, funding acquisition (supporting), project administration, resources, supervision (lead), writing – review and editing (equal). **M.K.:** methodology, supervision (supporting), visualization (supporting), writing – review and editing (equal).

## Conflicts of Interest

The authors declare no conflicts of interest.

## Supporting information


Table S1.

## Data Availability

The data that support the findings of this study are available from the corresponding author upon reasonable request.
