# Supplementary material for: The Effects of War‐Related Stress on Human Development: Differences in Body Proportions of Polish Women Born Before and During World War II
Source: Am J Hum Biol. 2024 Oct 30;37(1):e24175. doi: 10.1002/ajhb.24175 (PMC11669067; doi:10.1002/ajhb.24175)
Supplement: Supplementary file 1 — Table S1. [file AJHB-37-e24175-s001.pdf]

Supplementary Material

Response	Pre-war Group						War Group					
	Df	Sum Sq	Mean Sq	F value	p value		Df	Sum Sq	Mean Sq	F value	p value	
Body height												
Living Brothers	1	2424	2423.6	0.92	0.34		1	1025	1025.0	0.39	0.53	
Living Sisters	1	6139	6138.6	2.32	0.13		1	11540	11539.6	4.43	0.04	*
Living Brothers : Sisters	1	4526	4526.4	1.71	0.19		1	2997	2997.4	1.15	0.28	
Living Siblings	1	7295	7294.6	2.76	0.10	·	1	8843	8843.1	3.39	0.07	·
Deceased Brothers	1	561	560.8	0.22	0.64		1	0	0.3	0.00	0.99	
Deceased Sisters	1	16203	16203.1	6.31	0.01	*	1	1474	1473.9	0.55	0.46	
Deceased Brothers : Sisters	1	9816	9816.2	3.83	0.05	·	1	2055	2054.9	0.77	0.38	
Deceased Siblings	1	6136	6136.2	2.31	0.13		1	57	57.2	0.02	0.88	
Symphysion height												
Living Brothers	1	1519	1518.6	1.10	0.30		1	126	125.7	0.08	0.78	
Living Sisters	1	5316	5315.6	3.83	0.05	·	1	4928	4928.4	3.19	0.08	·
Living Brothers : Sisters	1	706	706.0	0.51	0.48		1	1843	1843.1	1.19	0.28	
Living Siblings	1	6115	6114.8	4.44	0.04	*	1	2728	2727.9	1.76	0.19	
Deceased Brothers	1	4520	4519.6	3.37	0.07	·	1	978	978.1	0.62	0.43	
Deceased Sisters	1	5890	5889.6	4.40	0.04	*	1	72	71.6	0.05	0.83	
Deceased Brothers : Sisters	1	5264	5263.8	3.93	0.05	*	1	235	235.1	0.15	0.70	
Deceased Siblings	1	8791	8790.6	6.45	0.01	*	1	1376	1376.0	0.88	0.35	
Biacromial Width												
Living Brothers	1	467	466.9	1.30	0.26		1	338	338.5	1.07	0.30	
Living Sisters	1	2	2.4	0.01	0.94		1	986	985.5	3.11	0.08	·
Living Brothers : Sisters	1	545	545.1	1.52	0.22		1	343	343.0	1.08	0.30	
Living Siblings	1	202	201.9	0.56	0.45		1	1061	1061.0	3.35	0.07	·
Deceased Brothers	1	156	155.8	0.44	0.51		1	120	120.3	0.37	0.54	
Deceased Sisters	1	1320	1319.6	3.72	0.06	·	1	185	184.8	0.57	0.45	
Deceased Brothers : Sisters	1	268	268.0	0.76	0.39		1	140	140.3	0.43	0.51	
Deceased Siblings	1	24	24.2	0.07	0.80		1	287	286.8	0.89	0.35	
Trunk Length												
Living Brothers	1	1694	1693.8	2.28	0.13		1	1420	1419.7	1.58	0.21	
Living Sisters	1	2336	2336.1	3.15	0.08	·	1	587	586.9	0.65	0.42	
Living Brothers : Sisters	1	1774	1774.0	2.39	0.12		1	147	147.4	0.16	0.69	
Living Siblings	1	3218	3218.1	4.30	0.04	*	1	2377	2377.2	2.68	0.10	
Deceased Brothers	1	2318	2317.9	3.05	0.08	·	1	396	396.1	0.44	0.51	
Deceased Sisters	1	43	43.2	0.06	0.81		1	752	752.1	0.83	0.36	
Deceased Brothers : Sisters	1	284	284.2	0.37	0.54		1	143	143.3	0.16	0.69	
Deceased Siblings	1	1694	1693.5	2.24	0.14		1	736	736.2	0.82	0.37	
Waist Circumference												
Living Brothers	1	842	842.5	0.51	0.48		1	5254	5253.9	3.60	0.06	·
Living Sisters	1	20264	20263.9	12.32	0.00	***	1	33	32.5	0.02	0.88	
Living Brothers : Sisters	1	0	0.2	0.00	0.99		1	9511	9511.4	6.51	0.01	*
Living Siblings	1	7510	7509.8	4.41	0.04	*	1	1556	1555.6	1.02	0.31	
Deceased Brothers	1	8091	8091.2	4.76	0.03	*	1	6579	6579.1	4.37	0.04	*
Deceased Sisters	1	1501	1501.2	0.88	0.35		1	379	379.2	0.25	0.62	
Deceased Brothers : Sisters	1	1567	1566.6	0.92	0.34		1	957	957.5	0.64	0.43	
Deceased Siblings	1	10150	10150.1	6.01	0.02	*	1	7266	7266.4	4.88	0.03	*

Table S1

Analysis of variance (ANOVA) for the number of living and deceased siblings influencing response variables (body measurements) among women from the pre-war and the war group. Note: The variables "living siblings" and "deceased siblings" represent the total number of living or deceased siblings, combining both brothers and sisters into one measure. In contrast, the interaction between brothers and sisters ("living brothers : sisters" and "deceased brothers : sisters") examines how the combined presence of different numbers of brothers and sisters influences the response variable. Significance codes: 0 '***' 0.001 '**' 0.01 '*' 0.05 '·' 0.1 ' ' 1.
